# Supplementary material for: How survey results are reported in the media: A framework on selection mechanisms and a pilot study on reporting practice
Source: PLoS One. 2025 Oct 8;20(10):e0333066. doi: 10.1371/journal.pone.0333066 (PMC12507245; doi:10.1371/journal.pone.0333066)
Supplement: Attachment — Submitted filename: Response to Reviewers.pdf [file pone.0333066.s001.pdf]

Response to Editor and Reviewers for Revised Article "The Impact of Statistical Literacy and Economic Incentives on the (Mis-)Use of Survey based Statistics in Media Reporting — A Framework"

July 27, 2025

We are indebted to the reviewers for reading again the revised version of our paper and the further comments received from one reviewer and the editor.

We are pleased that by our first revision we were able to address all points originally raised by reviewer #2 and also most of those indicated by reviewer #1. In the following, we will describe how the remaining minor comments of reviewer #1 and the helpful guidelines provided by the editor were addressed.

The original comment is repeated first, followed by our response in italics.

1 Reviewer #1

I am reviewing this manuscript for the second time. As I have already mentioned before: The topic and the research question addressed in the manuscript is of enormous relevance in a world in which more and more statistics are disseminated through the media and in which there is a great demand for statistics that are both good and well reported. I am very pleased that the authors have also provided a little empirical insight into articles in BILD. This extension is quite exciting and an enhancement of the article. Overall, I think that the revision of the article has been successful and I only have minor aspects that need to be revised, which I will list in the following:

We are glad that the reviewer appreciates our added analysis for BILD, which provides first insights into the heterogeneity that could be expected across different types of news outlets. It is good news for us that we could address all the major issues from her/his first report and did our best to deal with the remaining minor issues as described below.

Minor issues: 1) I believe that the authors are still not sufficiently aware of which and how many actors are involved in certain media articles and other media products and that these require a very different kind of statistical literacy.

The reviewer comes back to his argument that our framework does not cover all relevant actors and that the role of different actors might require different kinds of statistical literacy. We agree on these limitations and – also in accordance with comment 4) and a related comment by the editor tried to make them more transparent throughout the paper.

2) You provide a definition of "statistical literacy" very late in the article. Furthermore, there is the headline "Impact of statistical literacy" in where you first describe scientific literacy, which is quite confusing for readers. It would be better to refer to this section already when you first mention statistical literacy at page 3, line 92. Furthermore, the definition and explanation of statistical literacy is still weak. When reading your article, I sometimes thought about the quote from H.G. Wells, who said "Statistical thinking will one day be as necessary for efficient citizenship as the ability to read and write!"

We are indebted to the reviewer for this comment. We see the point that the first discussion of statistical literacy on page 3, line 92 (in the version with track changes) is not comprehensive enough. Thus, we follow the suggestion of adding a reference to the section 'Impact of Statistical Literacy', where a more comprehensive discussion is provided. In that section, we deliberately introduce both scientific and statistical literacy in order to distinguish between the two concepts, as we explicitly state. That said, we fully agree with the spirit of the H.G. Wells quote and appreciate its relevance.

3) Figure 1 contains markings from the grammar check. Please delete them.

Thanks for pointing out this shortcoming of the previous version, which has been corrected.

4) Line 202 "still contains markings from the grammar check. Please delete them." In sentences like this, it seems as if you have thought through all the groups of people in detail. But I think that's missing from the paper. If you do not fulfill this (as you write in the answer - which is also okay), please weaken these sentences accordingly.

The markups you mention show up in the revised manuscript with track changes, but not in the manuscript itself. They are shown in the version with track changes to highlight new parts and removed parts in addition to the different colors.

Your comment on the groups of people is related to comment 1) and similar to a statement made by the editor. Therefore, we checked all sentences regarding the groups of people considered in the framework and made adjustments to avoid the impression that the article provides a complete picture of all potentially relevant groups of people and a detailed description of all factors driving their decisions.

5) Line 404: "Statistical literacy may be essential for journalists when selecting high-quality survey results to report on, . . . " This is a very narrow view of statistical literacy for journalists. Statistical literacy is not only necessary for evaluating the quality of the studies.

We fully agree and have expanded the relevant section to highlight additional advantages statistical literacy offers journalists beyond evaluating study quality.

6) Line 505: 21. 89% Please delete the blank space.

Actually in line 555 of the revised version with track changes: Done.

7) Line 581: Please add an example for a statistical key word you mean here.

As described in the text, we identified various statistical keywords such as 'representative', 'error rate', 'sampling procedure', and others. For better clarity, we have now also included them in the description of the figure categories, as you suggested.

8) Figure 2: The percentage sign is not placed very well. Furthermore, it would be great to provide the full area to 100% (instead of 70%).

We changed the scale to 0 – 100%.

9) Line 626: It would be great to insert the 9 out of 144 also in Figure 2.

One way of including this information in Figure 2 would be to add an additional category. This would not change anything to the blue bars, but reduce the height all of remaining organge bars (dividing by 144 instead of by 135). However, since our focus is on the reporting on surveys, this might be misleading since the 9 articles do not refer to any specific survey. Therefore, we prefer to exclude these articles from the figure. However, the qualitative result would not be affected.

10) Line 628: Here you are speaking about red bars, whereas in Figure 2 you are speaking about orange bars.

Thanks for pointing out this error referring to an earlier version of the figure with red bars. We changed to "orange bars".

11) Line 650: Is the study really about a theoretical framework for survey reporting in the media? I think what you write in the footnote of Figure 1 matches better.

We agree that the presentation of the small-scale empirical pilot study might suggest a more narrow perspective. However, the focus of our paper is on a theoretical framework for survey reporting in the media. We clarified this throughout the text.

12) Line 651 "we have systematically analyzed the role of different actors": No you have not! Please delete "systematically"

In accordance with our reply to your points 1) and 4) we agree that the statement might be too strong. Therefore, we removed the word "systematically" as suggested.

13) Sometimes you speak about a pilot study, a small-scale and exploratory empirical pilot study, an exploratory small-scale pilot study. Please standardize throughout the article.

Thank you for pointing out the inconsistency. We have adjusted the formulation accordingly.

2 Editor

Please check capitalization of the title: The Impact of statistical Literacy and economic Incentives on the (Mis-)Use of Survey based Statistics in Media Reporting – A Framework

Done.

The Abstract It should include alternatives that are much more obvious for not reporting methodological background than economic incentives, e.g. the perception of journalists that the audience may be overstrained by that, or just space and time reasons, and collision with journalist principles like "pointing". The method of the study should appear there.

We have revised the abstract to include the additional reasons you suggested for omitting methodological details, and we have also referenced our small-scale empirical pilot study of BILD and DER SPIEGEL to support the relevance of our framework.

Introduction Please justify the focus on survey-based statistics clearly. Please justify the focus on types of media [social media?, bloggers?].

Thanks for pointing out that these aspects need justification. We clarified the focus on survey statistics and included an argument why we focus on mass media.

What is the point about "surprising" and "negative" for your argumentation?

Thank you for pointing this out. We agree that the notions of "surprising" and "negative" findings are not central to the current analysis. Although such aspects might be relevant in future empirical studies exploring how the framing or perceived news value of survey results influences media uptake, they are not further discussed in this paper. For this reason, we have removed the respective part of the paragraph to maintain a clear focus.

"In a limited sample of articles from the journal DER SPIEGEL and BILD..." Is this result or part of the argument for doing the study? Please elaborate the argument if studies are commissioned more explicitly.

Thank you for this helpful question. We have clarified our argument in the manuscript as to why it is relevant to examine the share of commissioned studies. We do not claim that commissioned studies are more likely to be covered in the media, as we do not have information on surveys that were not reported. However, we observe that a considerable share of the surveys in our sample were commissioned. Although we cannot draw causal conclusions from this, we have decided to keep this observation as it may be relevant for future research, particularly regarding the topics and contexts in which surveys are commissioned. We added a sentence in the introduction and mention the issue when discussing limitations to highlight that this finding of our empirical analysis deserves attention in future research.

What is Plan International? Consider taking the elaboration on the shortcomings of the Plan International Example as the starting point for outlining the motivation of the study - what are crucial methodological details that are omitted typically?

Thank you for this helpful comment. We have added a brief explanation of Plan International, including a link to the NGO in the manuscript to ensure clarity for all readers.
The findings of the Plan International study serve primarily to motivate our research, as they clearly illustrate that there are differences in the way surveys are reported in the media. However, this example is not particularly well suited to discuss methodological shortcomings in detail, beyond the issues we already mentioned, such as representativeness and selection bias. For this reason, we refer to it mainly in the context of media attention rather than

methodological critique.

The third question leaves the shortcomings of the example, but aims at the procedural level. Can you study the different levels at once?

We are not entirely sure what your comment refers to. Just to clarify, the third research question was: "Third, how were these results presented to readers and how much attention did they receive?" We do return to the example of the Plan International study, as it raised the question of why this particular survey received extensive media coverage, while official statistics on the same topic did not. Our theoretical framework highlights some factors that may influence whether and how surveys are reported in the media, such as funding sources or the speed of result availability. Although we cannot empirically determine the exact reasons <u>*why*</u> *certain surveys receive more attention than others, we can observe and analyze the fact* <u>*that*</u> *they were reported. We acknowledge that this limits our ability to study all levels simultaneously, but we believe that our approach offers valuable information on the dynamics of media coverage and survey visibility.*

"First, we describe the economic incentive structure for the whole pipeline from running a survey" If this is the goal, the motivation need to be sharpened. "Second, we also want to take into account the level of "statistical literacy" [of whom]?

We changed the description of the framework as one that defines the general actors involved in the production of mass media with a focus on the incentives in reporting the results of surveys by mass media. We specified that statistical literacy is relevant to media and users as actors.

The methodological section is crucial prior to your review - of the theoretical and empirical literature on economic incentives for journalists and newspapers. - of the role of statistical literacy for journalists

For the report on the empirical study, we introduced the section 'Methods' that describes the decision to use both outlets, the key methods of text selection, data cleaning, and analysis. Consequently, the results of the study are presented in the 'Results' section.

Why the focus on Germany? Why the focus on those two media outlets?

We focus on Germany to examine the behavior of the media in a high-income democracy with strong media institutions. BILD and DER SPIEGEL were selected due to their wide reach and contrasting editorial styles, which allow comparative insight into survey reporting practices. We discuss this in detail in 'Methods' (p. 10ff), where we outline the relevance of BILD and SPIEGEL for our research focus. As noted in the final paragraph of the paper, we would very much like to extend our analysis to additional media outlets and countries in the future, once this can be done using automated methods rather than manual coding.

Fig.1 should avoid correction marks included.

Done following also the recommendation by Reviewer #1.

Fig.2: capitalization, uncertainty information is missing, axis is not in the PDF, axis label is missing,

We assume that "capitalization" refers to the labels of the two newspapers. In fact, both newspapers use capitalization as part of the brand. Therefore, we also use it both in the text and in Figure 2, whenever referring to them.
We made the following changes to the figure: added error bars indicating +/- one standard deviation for the shares, modified the y-axis to 0 - 100 % as suggested by reviewer #1 and added axis arrows and axis labels.

The results section should be clearly marked as such. The discussion is missing and possibly mixed with implications. Limitations of the current study are important.

We included the 'Results' section. We renamed the 'Implications' into 'Discussion', in which the limitations of the current study are stated more clearly.
