# Supplementary material for: How survey results are reported in the media: A framework on selection mechanisms and a pilot study on reporting practice
Source: PLoS One. 2025 Oct 8;20(10):e0333066. doi: 10.1371/journal.pone.0333066 (PMC12507245; doi:10.1371/journal.pone.0333066)
Supplement: Attachment — Submitted filename: Response.pdf [file pone.0333066.s002.pdf]

Response to Editor for 3rd Revision of Article "How survey results are reported in the media: A framework on selection mechanisms and a pilot study on reporting practice", formerly titled "The Impact of Statistical Literacy and Economic Incentives on the (Mis-)Use of Survey based Statistics in Media Reporting — A Framework"

September 3, 2025

We appreciate that the editor acknowledges that our second revision could deal with the comments from one reviewer and himself in an adequate way. In the present final revision, we address the following minor modifications recommended by the editor:

The original recommendation is repeated first, followed by our response in italics.

– Method and Results have to be written in past tense (see the abstract).

  We changed the description of Methods and Results to past tense throughout the paper including the relevant sentences in the abstract.

– Rethink the title please, because it should be much closer to the actual insight that your method allowed to investigate.

  Based on an earlier comment from one of the reviewers, we already thought about alternative titles that fit better the actual content of the article. We propose the following modified title:

  "How survey results are reported in the media: A framework on selection mechanisms and a pilot study on reporting practices" or, if you prefer a more concise title, "Survey reporting in the media: A framework and a pilot study"

– Your justification for choosing „mass media" evokes the question for the underlying definition of mass media – for instance you indicate they are considered „high-quality media" – do you understand mass media as equaling the „leading media", the traditional media.

  In this paper, we use the term "mass media" to refer to traditional, widely circulated news outlets with large audiences and significant agenda-setting power. This includes

both high-quality investigative journalism (e.g., DER SPIEGEL) and more populist formats (e.g., BILD). While these outlets differ in tone and editorial standards, they share the characteristic of reaching mass audiences through institutionalized journalistic practices.

We changed the wording from "the mass media is recognized as high-quality media, relevant for the formation of opinions of citizens" to "the mass media are recognized as widely circulated news outlets with large audiences and significant agenda-setting power, relevant for the formation of opinions of citizens".

- Introduction "In a limited sample of articles from the journal DER SPIEGEL and BILD..." – you should refrain from placing results in the introduction, where you motivate the study producing the results

  As suggested we removed the part presenting results in the introduction.

- Capitalization should be consistent across all terms in all figures (e.g., perfect in Figure 1).

  We adjusted capitalization to use capital letters only for the first term in each box, for each axis etc.
